# Supplementary material for: Sleep maintains excitatory synapse diversity in the cortex and hippocampus
Source: Curr Biol. 2024 Aug 19;34(16):3836–3843.e5. doi: 10.1016/j.cub.2024.07.032 (PMC11359089; doi:10.1016/j.cub.2024.07.032)
Supplement: Document S1. Figures S1–S4 [file mmc1.pdf]

Current Biology, Volume *34*

Supplemental Information

Sleep maintains excitatory synapse diversity in the cortex and hippocampus

Dimitra Koukaroudi, Zhen Qiu, Erik Fransén, Ragini Gokhale, Edita Bulovaite, Noboru H. Komiyama, Julie Seibt, and Seth G.N. Grant

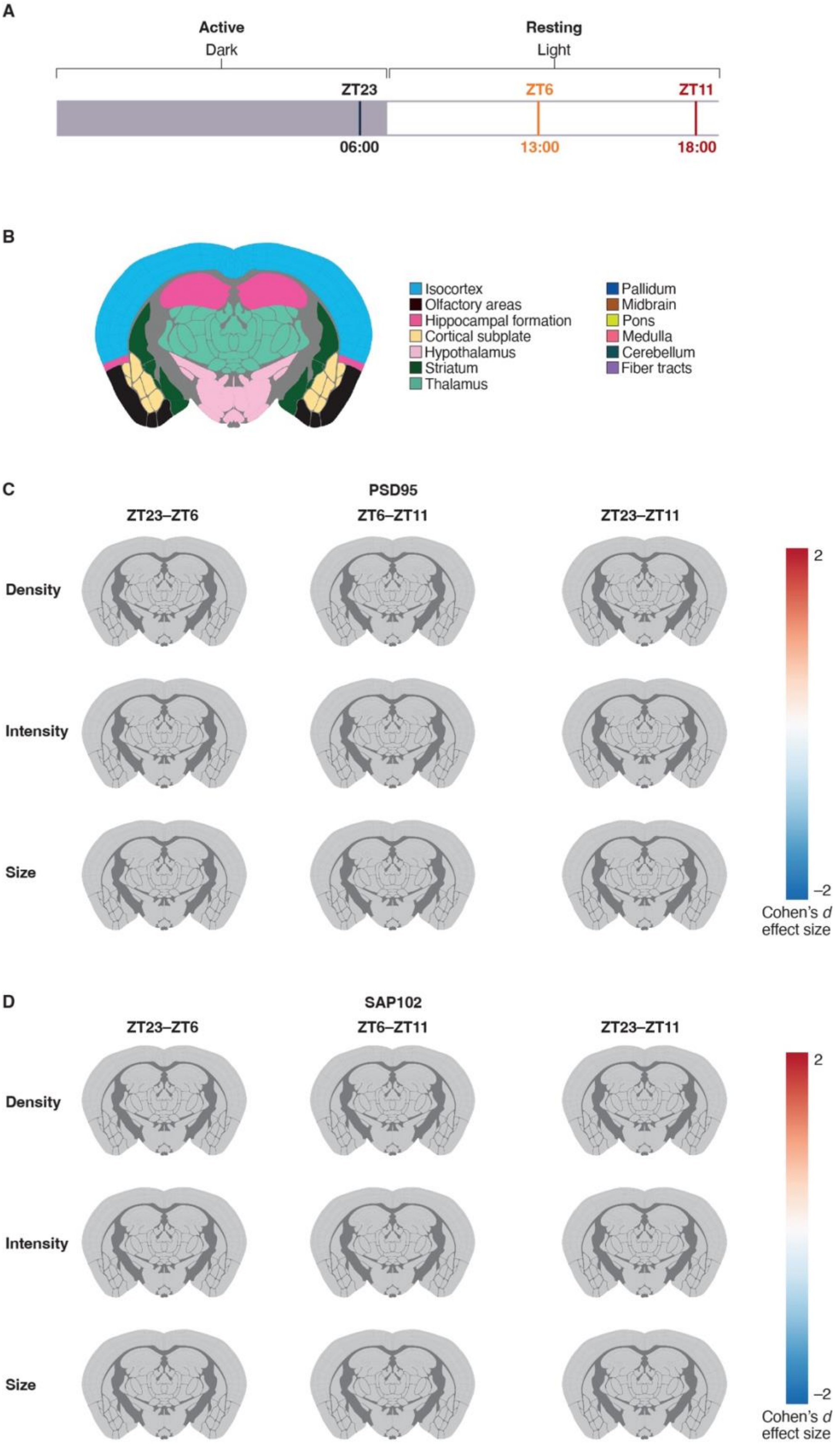
A
Active
Dark
Resting
Light
ZT23
ZT6
ZT11
06:00
13:00
18:00
B
Isocortex
Olfactory areas
Hippocampal formation
Cortical subplate
Hypothalamus
Striatum
Thalamus
Pallidum
Midbrain
Pons
Medulla
Cerebellum
Fiber tracts
C
PSD95
ZT23–ZT6
ZT6–ZT11
ZT23–ZT11
Density
Intensity
Size
2
−2
Cohen's d effect size
D
SAP102
ZT23–ZT6
ZT6–ZT11
ZT23–ZT11
Density
Intensity
Size
2
−2
Cohen's d effect size

Figure S1 The synaptome architecture of PSD95 and SAP102 puncta does not change in the circadian cycle. Related to Figure 2.

(A) Schematic representation of light/dark cycle, with zeitgeiber time (ZT) showing when mice were sampled (ZT23, ZT6, ZT11) and corresponding 24-hour clock (06:00, 13:00, 18:00). (B) Key for brain regions shown in maps (C,D). (C)Synaptome maps of PSD95 puncta density (top row), intensity (middle row), size (bottom row) changes for periods ZT23-ZT6 (left column), ZT6-ZT11 (middle column), ZT23-ZT11 (right column) in brain regions shows no significant changes (grey; P>0.05, Bayesian test with Benjamini-Hochberg correction). (D) Synaptome maps of SAP102 puncta density (top row), intensity (middle row), size (bottom row) changes for periods ZT23-ZT6 (left column), ZT6-ZT11 (middle column), ZT23-ZT11 (right column) in brain regions shows no significant changes (grey; P>0.05, Bayesian test with Benjamini-Hochberg correction).

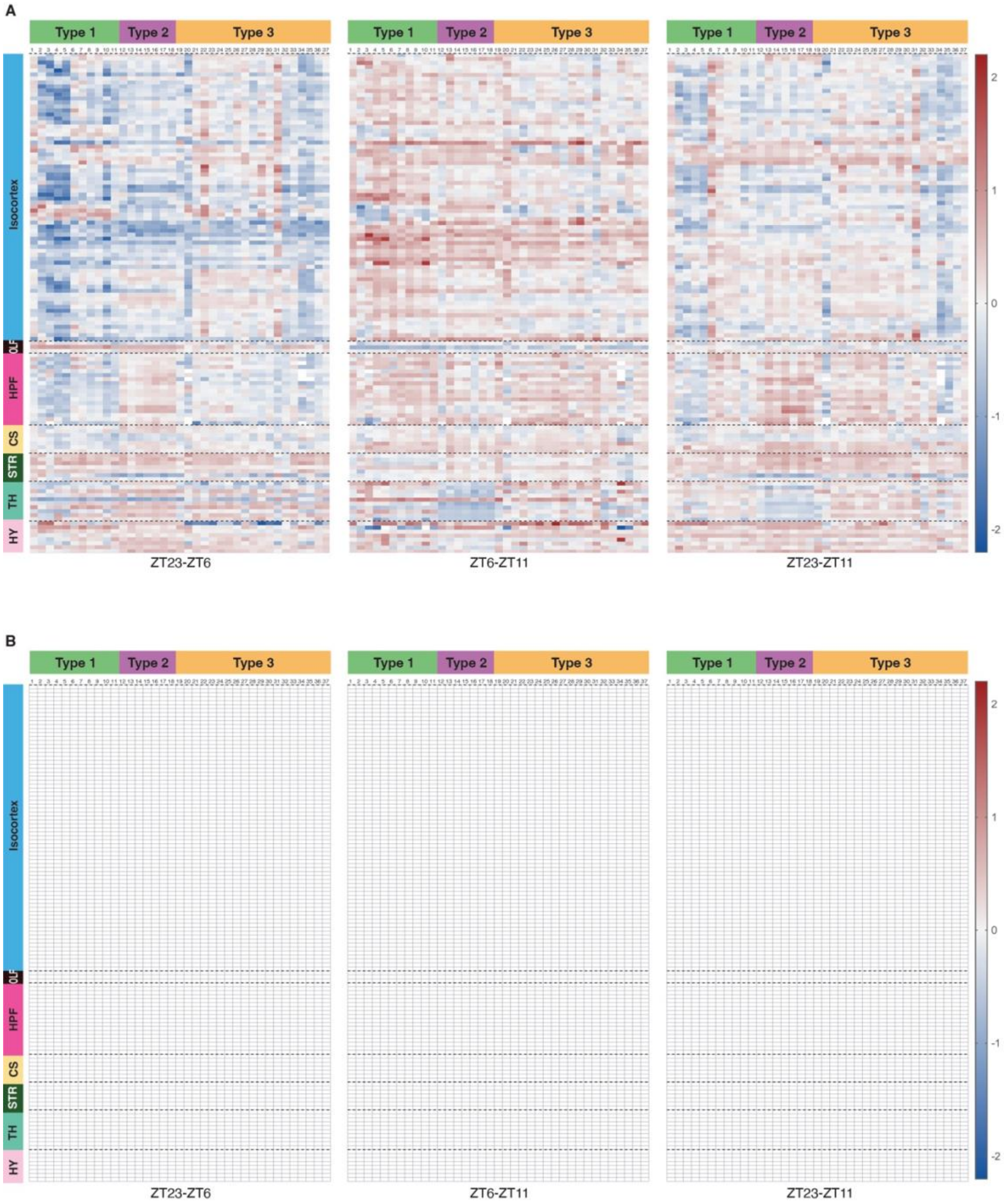

Figure S2 The synaptome architecture of Type 1, Type 2 and Type 3 synapse subtypes in the circadian cycle. Related to Figure 2.

Heatmaps of SD-induced changes in the density (Cohen's d) of synapse subtypes in brain regions for periods ZT23-ZT6 (left panel), ZT6-ZT11 (middle panel), ZT23-ZT11 (right panel). (A) raw values, (B) significant values after multiple correction testing (Bayesian test with Benjamini-Hochberg correction). All datapoints show P>0.05. Regions; isocortex; OLF, olfactory areas; HPF, hippocampal formation; CS, cortical subplate; STR, striatum; TH, thalamus; HY, hypothalamus.

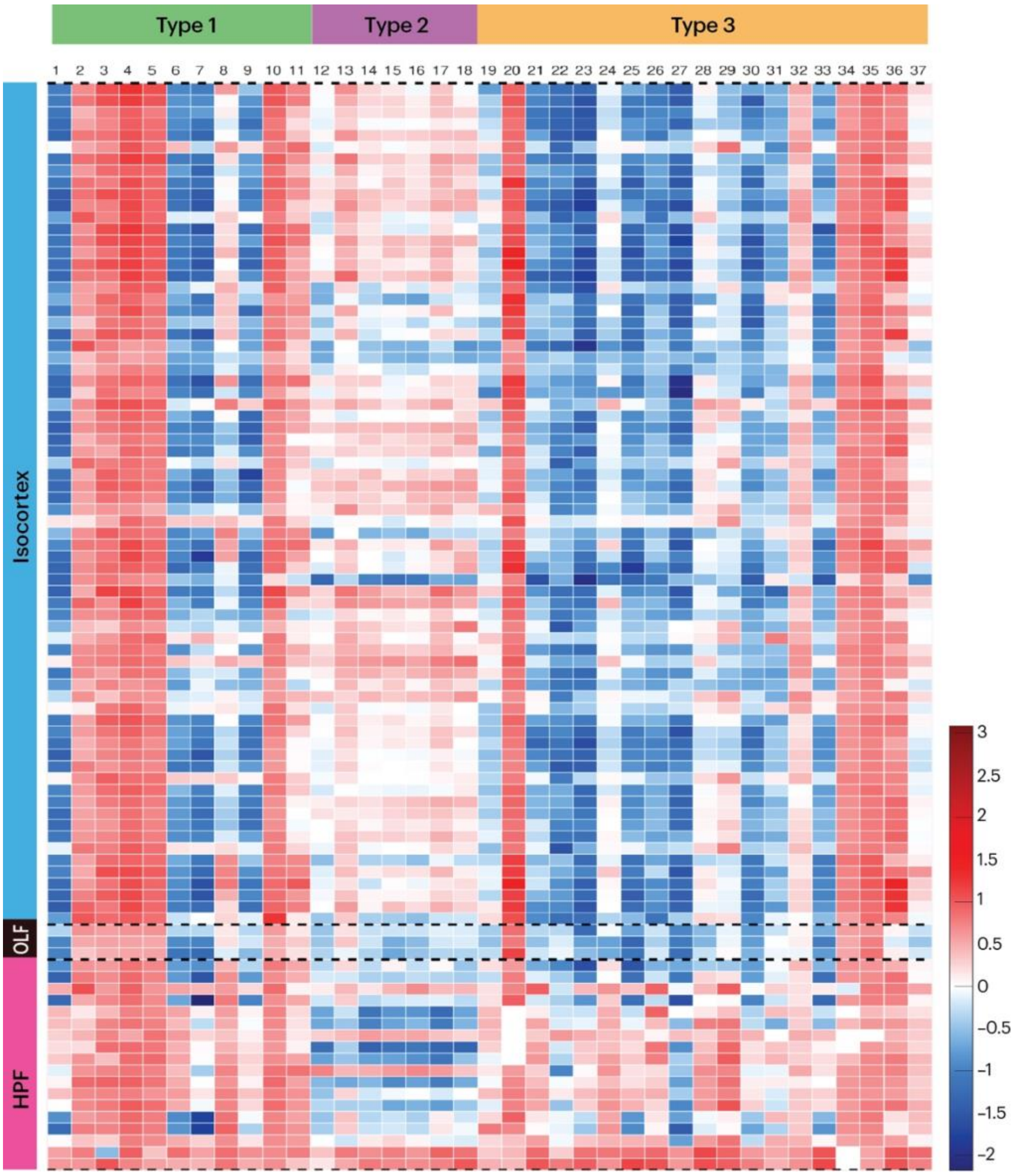

Figure S3. SD differentially impacts synapse subtypes. Related to Figures 2, 3. Heatmap of SD-induced changes in the density (Cohen's d) of synapse types and subtypes. Uncorrected. Significant changes are shown in Figure S4. Regions: isocortex; OLF, olfactory regions; HPF, hippocampal formation.

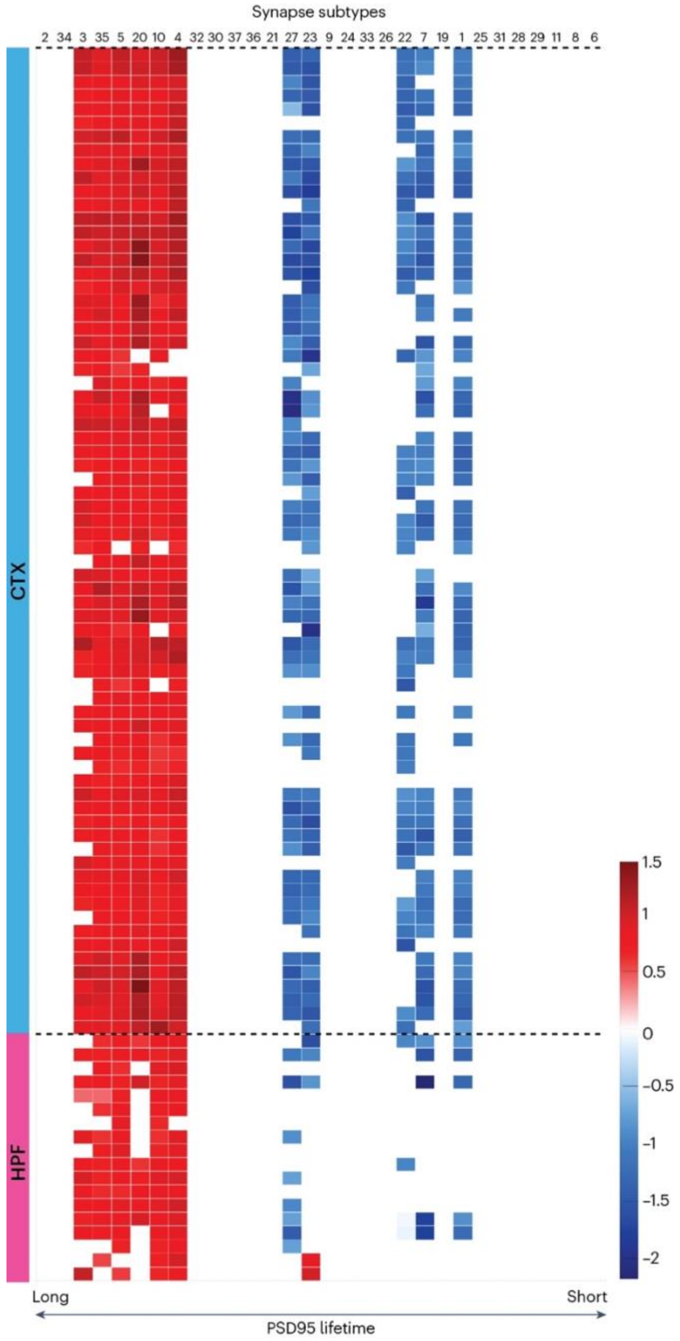

Figure S4. SD differentially impacts synapse subtypes with long and short protein lifetimes. Related to Figures 2, 3.

Heatmap of SD-induced changes in the density (Cohen's d) of synapse subtypes ranked from longest to shortest PSD95 lifetime[S1]. Significant changes are shown ($P<0.05$, Bayesian test with Benjamini-Hochberg correction).

Supplemental reference

S1. Bulovaite, E., Qiu, Z., Kratschke, M., Zgraj, A., Fricker, D.G., Tuck, E.J., Gokhale, R., Koniaris, B., Jami, S.A., Merino-Serrais, P., et al. (2022). A brain atlas of synapse protein lifetime across the mouse lifespan. Neuron *110*, 4057-4073 e4058.
