# Supplementary material for: Sleep maintains excitatory synapse diversity in the cortex and hippocampus
Source: Curr Biol. 2024 Aug 19;34(16):3836–3843.e5. doi: 10.1016/j.cub.2024.07.032 (PMC11359089; doi:10.1016/j.cub.2024.07.032)
Supplement: Document S2. Article plus supplemental information [file mmc2.pdf]

Report

Current Biology

Sleep maintains excitatory synapse diversity in the cortex and hippocampus

Graphical abstract

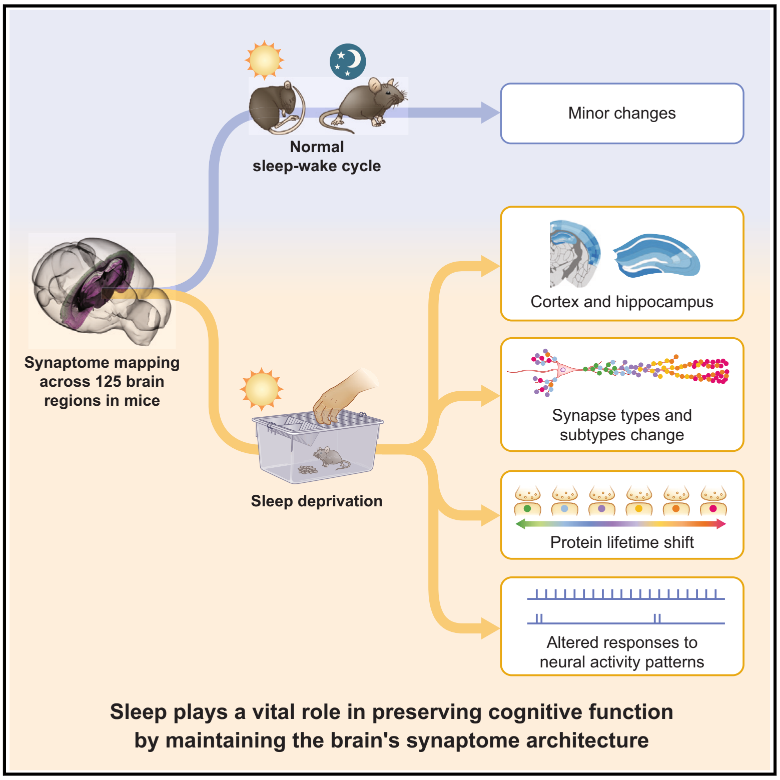

Highlights

- Lack of sleep alters brain synaptome architecture within hours
- Sleep deprivation reduces synapse diversity in the cortex and hippocampus
- Sleep deprivation impacts specific molecular types and subtypes of synapses
- Synapses with long protein lifetimes are resilient to sleep deprivation

Authors

Dimitra Koukaroudi, Zhen Qiu,
Erik Fransén, ..., Noboru H. Komiyama,
Julie Seibt, Seth G.N. Grant

Correspondence

seth.grant@ed.ac.uk

In brief

Koukaroudi et al. show that sleep loss rapidly alters the synaptome architecture in areas of the brain that are crucial for memory and cognition. Preserving synapse diversity may be a fundamental role of sleep, helping to preserve normal brain function and mental health.

Koukaroudi et al., 2024, Current Biology *34*, 3836–3843
August 19, 2024 © 2024 The Author(s). Published by Elsevier Inc.
https://doi.org/10.1016/j.cub.2024.07.032

CellPress

CellPress
OPEN ACCESS

Current Biology

Report

Sleep maintains excitatory synapse diversity in the cortex and hippocampus

Dimitra Koukaroudi,[1] Zhen Qiu,[1,2] Erik Fransén,[3,4] Ragini Gokhale,[1] Edita Bulovaite,[1] Noboru H. Komiyama,[1,5,6,7] Julie Seibt,[8] and Seth G.N. Grant[1,5,9,*]
[1]Genes to Cognition Program, Centre for Clinical Brain Sciences, University of Edinburgh, Edinburgh EH16 4SB, UK
[2]School of Science and Engineering, University of Dundee, Dundee DD1 4HN, UK
[3]Department of Computational Science and Technology, School of Electrical Engineering and Computer Science, KTH Royal Institute of Technology, 10044 Stockholm, Sweden
[4]Science for Life Laboratory, KTH Royal Institute of Technology, 171 65 Solna, Sweden
[5]Simons Initiative for the Developing Brain (SIDB), Centre for Discovery Brain Sciences, University of Edinburgh, Edinburgh EH8 9XD, UK
[6]The Patrick Wild Centre for Research into Autism, Fragile X Syndrome & Intellectual Disabilities, Centre for Discovery Brain Sciences, University of Edinburgh, Edinburgh EH8 9XD, UK
[7]Muir Maxwell Epilepsy Centre, University of Edinburgh, Edinburgh EH8 9XD, UK
[8]Surrey Sleep Research Centre, School of Biosciences, University of Surrey, Guildford, Surrey GU2 7XP, UK
[9]Lead contact
*Correspondence: seth.grant@ed.ac.uk
https://doi.org/10.1016/j.cub.2024.07.032

SUMMARY

Insufficient sleep is a global problem with serious consequences for cognition and mental health.[1] Synapses play a central role in many aspects of cognition, including the crucial function of memory consolidation during sleep.[2] Interference with the normal expression or function of synapse proteins is a cause of cognitive, mood, and other behavioral problems in over 130 brain disorders.[3] Sleep deprivation (SD) has also been reported to alter synapse protein composition and synapse number, although with conflicting results.[4–7] In our study, we conducted synaptome mapping of excitatory synapses in 125 regions of the mouse brain and found that sleep deprivation selectively reduces synapse diversity in the cortex and in the CA1 region of the hippocampus. Sleep deprivation targeted specific types and subtypes of excitatory synapses while maintaining total synapse density (synapse number/area). Synapse subtypes with longer protein lifetimes exhibited resilience to sleep deprivation, similar to observations in aging and genetic perturbations. Moreover, the altered synaptome architecture affected the responses to neural oscillations, suggesting that sleep plays a vital role in preserving cognitive function by maintaining the brain's synaptome architecture.

RESULTS

To better understand the role of sleep in regulating synapse protein composition and synapse number, we have employed synaptome mapping technology to uncover the effects of sleep deprivation (SD) on the mouse brain. Synaptome mapping enables highly systematic and large-scale analysis of the protein composition, protein lifetime, and morphology of billions of individual synapses on a brain-wide scale.[8–11] This approach has revealed that excitatory synapses are highly diverse and can be categorized into different types and subtypes that together comprise the "synaptome" of the brain.[10] These varieties of synapses are spatially distributed across all areas of the brain, forming the "synaptome architecture" (SA), which changes with age and disease.[8–11] The diversity of synapse types and subtypes increases dramatically during mouse development and, after stabilizing in young adults, gradually reduces with aging, with preferential preservation of synapses with the longest protein lifetimes.[8,11]

When the SA of a neuron or brain region receives patterns of neural activity, it produces a spatiotemporal physiological response that is governed by the protein composition of its synapses.[8–10,12] Thus, changes in the SA during development, aging, or disease can impact cognition. It is not known whether the synaptome and SA of excitatory synapses change during the normal sleep-wake cycle or whether they are affected by SD. Addressing these questions may shed light on why sleep is important for cognitive function and enhance our understanding of the biological mechanisms that control synapse diversity.

Synaptome mapping across the circadian cycle

Synaptome mapping of excitatory synapses in the brain utilizes a line of mice that express fluorescently labeled endogenous postsynaptic proteins PSD95 and SAP102[10] (Figure 1). These are scaffold proteins that assemble physically distinct multiprotein complexes[13] and play a crucial role in synaptic transmission, synaptic plasticity, and cognition.[14–16] Brain tissue sections from these mice were imaged in the mid-coronal plane at single-synapse resolution using high-throughput spinning disk microscopy with an optical resolution of approximately 270 nm. Our bespoke synaptome mapping pipeline[10] was applied to detect and segment billions of individual synaptic puncta. We

This is an open access article under the CC BY license (http://creativecommons.org/licenses/by/4.0/).

CellPress
OPEN ACCESS

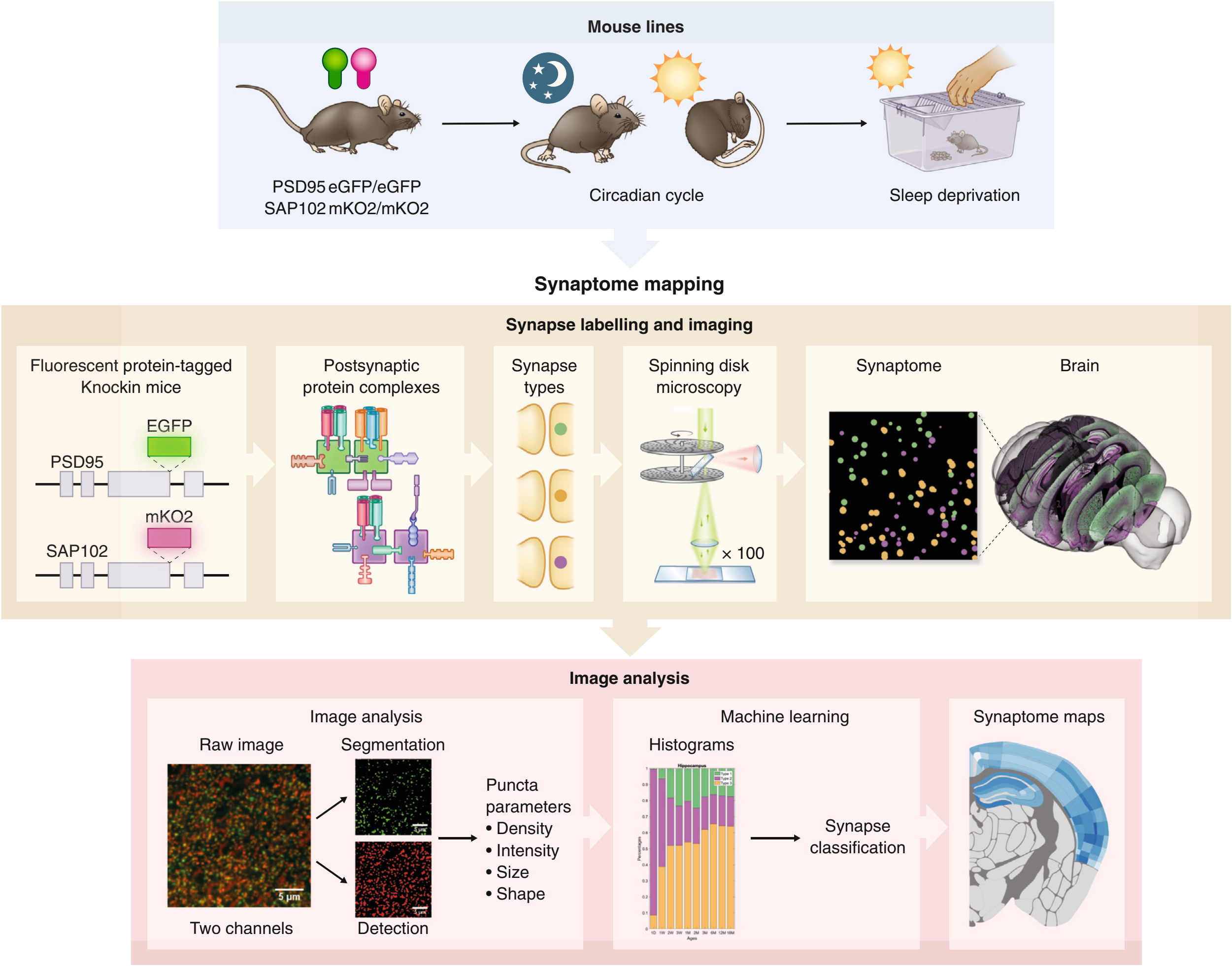

Figure 1. Workflow for synaptome mapping

Brain tissue from mice expressing the fluorescent proteins GFP and mKO2 fused to PSD95 and SAP102, respectively, was collected from the mice at different stages of the circadian cycle or after sleep deprivation. Synapse labeling and imaging shows genetic modification of PSD95 with eGFP and SAP102 with mKO2, which labels the proteins and their respective multiprotein complexes, which are distributed into synapse types/subtypes that can be visualized in brain sections using confocal spinning disc microscopy. The image analysis pipeline detects, segments, classifies, and quantifies synapse puncta, which are categorized into types and subtypes by machine learning and the spatial distribution plotted in synaptome maps.

measured the density, intensity, size, shape, and colocalization parameters of each punctum. Based on these parameters, we classified each synapse into one of three main types (type 1, PSD95 only; type 2, SAP102 only; type 3, PSD95 and SAP102) and further divided them into 37 subtypes using established synapse catalogs and machine learning methods.[8–10] Finally, we spatially mapped all the measurements of individual puncta and subtypes to construct a global synaptic atlas across 125 brain regions in mice at stages throughout the normal sleep-wake cycle and after SD.

As a first step toward understanding the role of sleep in the organization of the SA of the brain, we asked if there were any changes in the synaptome and SA during the normal circadian sleep-wake cycle. We compared three time points: the end of the active phase (zeitgeber time [ZT] 23) and the end (ZT11) and middle (ZT6) of the resting phase. We found no differences between these time points in any of the synapse parameters or in the density of synapse types and subtypes measured in any brain area ($p > 0.05$, Bayesian test with Benjamini-Hochberg correction) (Figure S1), indicating that the synaptome and the SA are stable throughout the normal sleep-wake cycle. Next, we examined the changes in the three types and 37 subtypes of excitatory synapses and observed minor changes in density between the three time points, but these changes were not statistically significant after correction for multiple testing ($p > 0.05$, Bayesian test with Benjamini-Hochberg correction) (Figure S2).

Sleep deprivation modifies the synaptome architecture

We next asked if SD induces any changes in the synaptome and SA. We compared mice after 6 h of SD (ZT6SD) with controls that were undisturbed over the same period (ZT6). SD did not affect the density or median intensity values of PSD95-expressing and/or SAP102-expressing synapses in any of the 125 brain

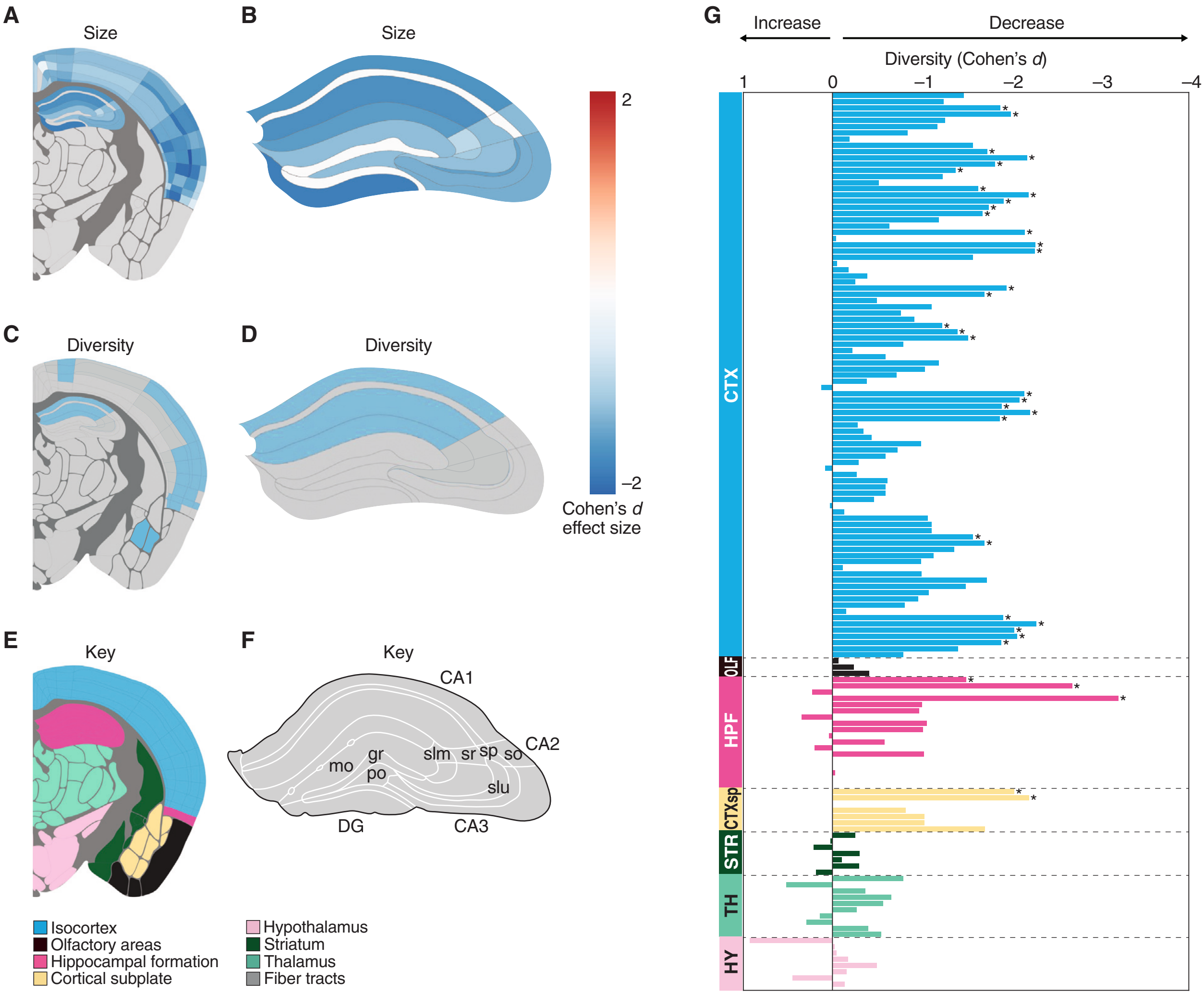

Figure 2. SD alters brain synaptome architecture within the cortex and HPF

SD reduces the size of PSD95-expressing synapses in cortical (A) and HPF (A and B) subregions. SD reduces synapse subtype diversity in cortical (C and G) and HPF (C, D, and G) subregions. Blue regions (A–D), Cohen's d effect size for regions with significant changes shown ($p < 0.05$, Bayesian test with Benjamini-Hochberg correction); gray regions (A–D), not significantly altered. (G) X axis shows change (Cohen's d) in synaptome diversity induced by SD. Asterisks indicate significant changes ($p < 0.05$, Bayesian test with Benjamini-Hochberg correction) in the diversity. Key for brain regions (E and F). Regions: CTX, isocortex; OLF, olfactory areas; HPF, hippocampal formation; CTXsp, cortical subplate; STR, striatum; TH, thalamus; HY, hypothalamus.
Related to Figures S1–S4.

subregions examined ($p > 0.05$, Bayesian test with Benjamini-Hochberg correction). However, SD did cause a decrease in the median size of synapses expressing PSD95 ($p < 0.05$, Bayesian test with Benjamini-Hochberg correction) (Figures 2A and 2B) but not SAP102 ($p > 0.05$, Bayesian test with Benjamini-Hochberg correction). The synaptome map of PSD95 puncta size showed that 99% (67/68) of cortical and 87% (20/23) of hippocampal formation (HPF) subregions were affected by SD ($p < 0.05$, Bayesian test with Benjamini-Hochberg correction), whereas subregions in other brain areas were unaffected ($p > 0.05$, Bayesian test with Benjamini-Hochberg correction). These results indicate that SD selectively modifies PSD95-expressing synapse types in the cortex and HPF, which are regions crucial for learning, memory, and sleep consolidation.[2]

Next, we investigated whether SD influences the high synapse diversity characteristic of the cortex and HPF regions.[8,10] By calculating the diversity of excitatory synapses based on the densities of 37 synapse subtypes, we found a significant reduction in synapse diversity across subregions of the cortex and the CA1 region of the HPF in response to SD (Figures 2C, 2D, and 2G). It was notable that the effect size in many regions was large, with a Cohen's d of around −2. To better understand how this reduction in synapse diversity was reflected among the 37 synapse subtypes, we created a heatmap of the density change (Cohen's d) for each subtype in the cortex and HPF (Figure S3). This shows that the density of some subtypes was consistently reduced across these regions, whereas the density in other regions was simultaneously increased, indicating that SD drives changes in the same subtypes in different neuron types.

CellPress
OPEN ACCESS

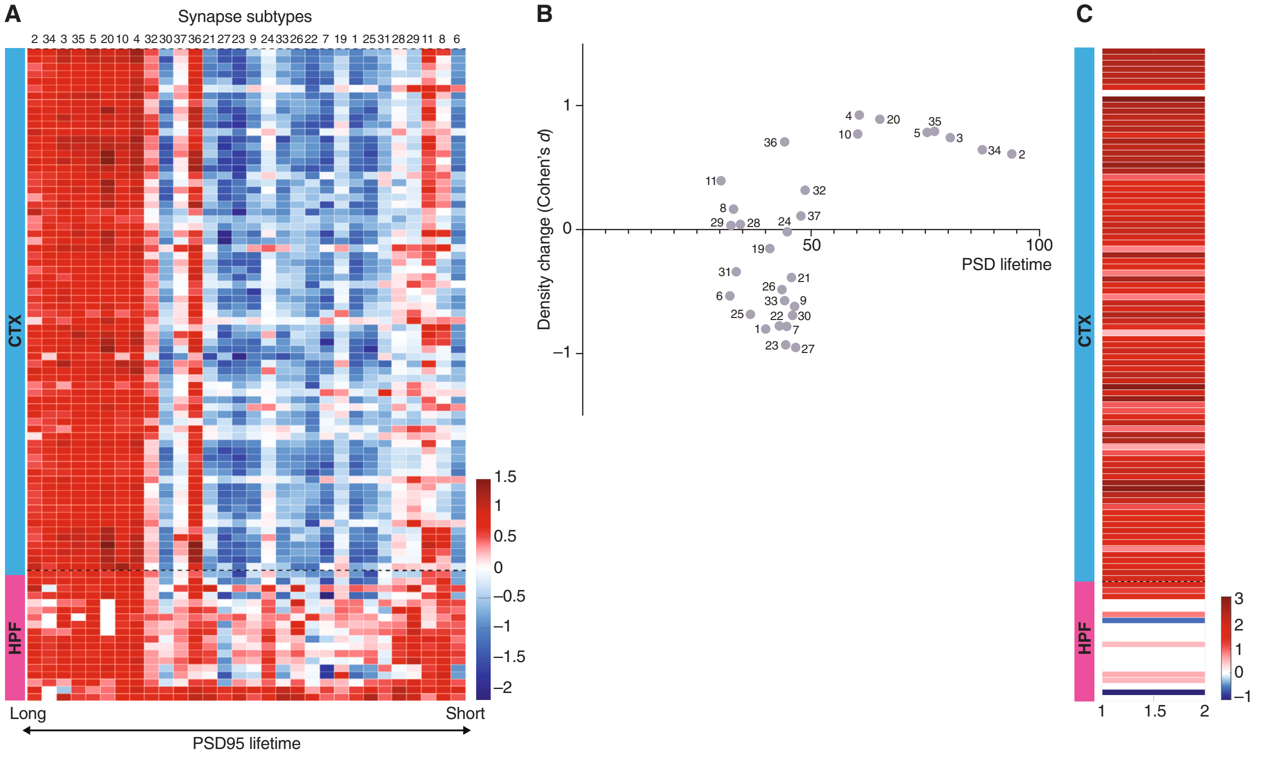

Figure 3. SD differentially impacts synapse subtypes

(A) Heatmap of SD-induced changes in the density (Cohen's d) of synapse subtypes in cortex (CTX) and HPF ranked from longest to shortest PSD95 lifetime.[11] For a heatmap showing significant changes corrected for multiple testing, see Figure S4.

(B) SD-induced synapse subtype density changes in the cortex (average of all subregions) plotted against PSD95 lifetime (normalized percentage).[11]

(C) Comparison of the density changes of six LPL (2, 3, 5, 20, 34, 35) with six SPL (6, 8, 11, 28, 29, 31) subtypes after SD. Red signifies subregions with greater change (Cohen's d) in LPL than SPL synapses, whereas blue signifies subregions with greater change in SPL than LPL synapses; white subregions show no significant differences.

Related to Figures S3 and S4.

Furthermore, because there is no net change in synapse density, the subtypes that are reduced in density may have transformed into those subtypes that are increased in density.

In previous studies, we discovered that certain "short protein lifetime" (SPL) subtypes of excitatory synapses exhibit faster turnover rates for PSD95 than others.[11] These SPL subtypes were involved in synaptic adaptations to mutations and aging.[9,11] By contrast, other "long protein lifetime" (LPL) subtypes, with a slower rate of PSD95 turnover, were selectively preserved in older individuals and were suggested to play a role in long-term memory storage.[8] Based on these findings, we hypothesized that the synapse subtypes most likely to undergo changes in response to SD would be those previously demonstrated under aging and mutation challenges to exhibit higher adaptability. Supporting our hypothesis, a heatmap representing the change in density of subtypes—ranked by their protein lifetime—across all regions of the cortex and HPF indicates that subtypes with longer protein lifetimes generally increased, whereas those with shorter lifetimes mostly decreased (Figures 3A and S4). Additionally, the relationship between the lifetime of PSD95 protein and the change in density (Cohen's d) of each subtype in the cortex reveals that synapses with shorter protein lifetimes experienced a decrease, whereas those with longer protein lifetimes showed an increase (Figure 3B). To further examine this, we compared the density changes between subtypes with the longest and shortest protein lifetimes. This revealed that the subtypes with longer lifetimes exhibited significantly greater increases than those with shorter lifetimes in various cortical subregions and select HPF subregions ($p < 0.05$, Bayesian test with Benjamini-Hochberg correction) (Figure 3C). In summary, our findings suggest that SD leads to a reduction in the diversity of excitatory synapses overall, primarily driven by a decrease in the number of synapse subtypes with short protein lifetimes and an increase in those with long protein lifetimes.

Sleep deprivation alters responses to patterns of neural activity in a computational model

Distinct patterns of neural activity are recognized as a defining characteristic of sleep and wakefulness, and it is widely believed that these patterns contribute to the process of memory encoding.[11] The SA plays a crucial role in converting patterns of neuronal activity into a spatiotemporal output,[10,12] and this process can be modified when there are changes in the SA.[8–10] To investigate whether SD-induced changes in the SA of the CA1 stratum radiatum (CA1sr) in the HPF could affect responses to activity patterns related to sleep, wakefulness, and memory

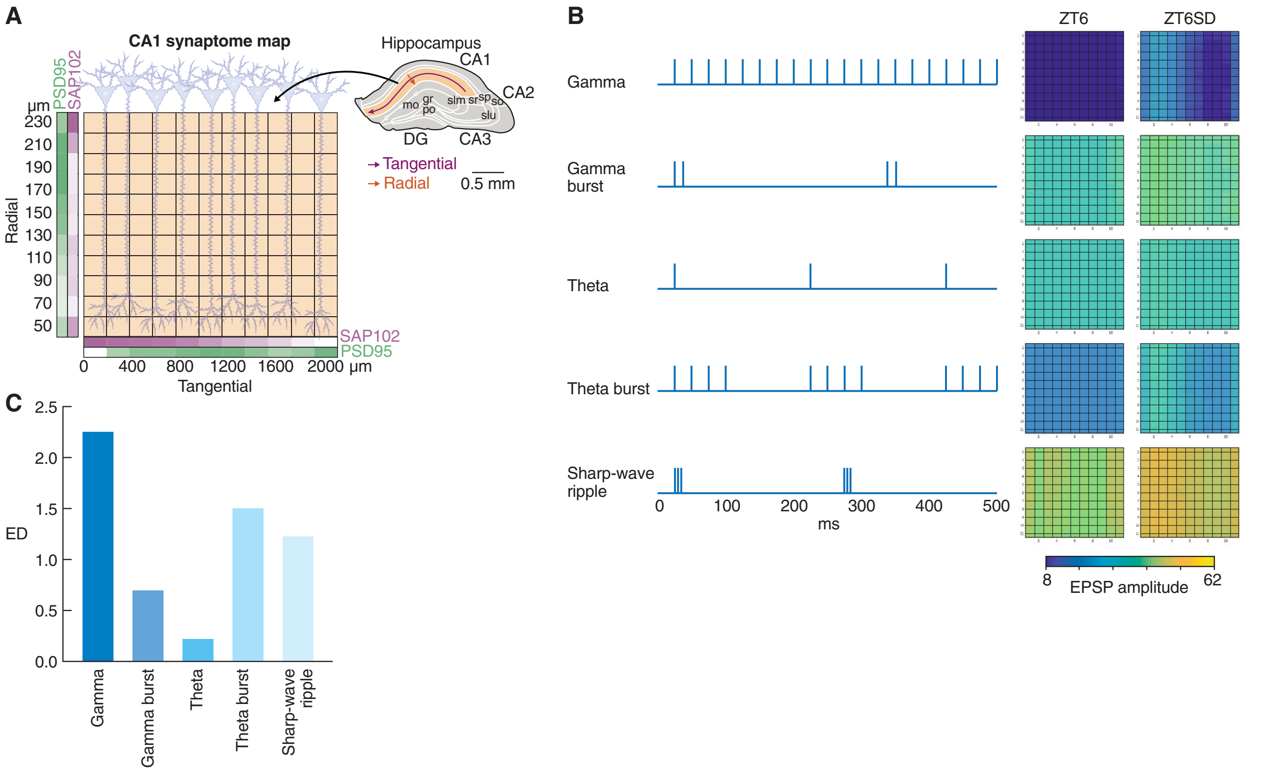

Figure 4. Computational modeling of physiological responses in the CA1sr to patterns of activity after SD

(A) The model simulates a 2D (11 × 11) array of synapses (boxes) expressing PSD95 and SAP102 measured along the radial and tangential axes of the CA1sr.[9,10]

(B) Five patterns of neuronal activity were used for CA1sr stimulation in the computational model. The summed excitatory postsynaptic potential (EPSP) response amplitudes in the ZT6 and ZT6SD groups were quantified (color bar, arbitrary units).

(C) Comparison of the extent of disruption caused by SD for each of the five patterns of activity. ED, Euclidian distance.

encoding, we employed a well-established computational model[8–10] (Figure 4A). When stimulating the CA1sr with five distinct activity patterns (gamma train, gamma burst, theta train, theta burst, sharp-wave ripple), each pattern had a different effect on SD versus control (p = 0.01, paired Kolmogorov-Smirnov test, n = 121; Benjamini-Hochberg corrected, n = 5; Cohen's d > 1.9) (Figure 4B). Notably, the most pronounced effects were observed in spatial responses for theta burst and gamma train patterns, while the intensity of responses was primarily influenced by the sharp-wave ripple pattern (Figures 4B and 4C).

DISCUSSION

The adult brain contains a vast number of excitatory synapses, which are highly diverse at the molecular level. This diversity arises and increases during postnatal development and reaches its highest levels in the cortex and HPF, where it is postulated to contribute to higher cognitive functions.[8,10] Our findings highlight the significance of sleep in preserving synaptome diversity in the cortex and HPF in the adult. By preserving synaptome diversity, sleep may contribute to optimizing cognitive function on a daily basis.

SD targets synapses with different rates of proteostasis

The synaptome of excitatory synapses comprises molecular types and subtypes of synapses, and we found that with 6 h of SD, the densities (number of synapses/area) of these types and subtypes were differentially affected without affecting the overall synapse density. These changes were most marked in the cortex and the CA1 regions of the HPF. Further inspection of these differential changes revealed that some subtypes of PSD95-expressing synapses were reduced, and others increased. Strikingly, this shift in the populations of PSD95-expressing subtypes corresponded with the protein lifetime of PSD95 in these subtypes, with the shift increasing the density of LPL synapse subtypes. A similar shift was previously found in the aging brain and was associated with a slowing of the rate of protein turnover.[8] Our findings are relevant to previous studies that show SD induces a slowing of protein synthesis which would be expected to result in slowing turnover.[17,18] Together, these observations suggest that SD modifies the SA through regulation of protein translation and turnover.

The slower rate of protein turnover in LPL synapses may render the sleep-deprived and aged brain less adaptable, potentially impairing learning and repair. Consistent with this, the repair of the SA in *Pax6* mice during development is primarily mediated by SPL synapses.[9] Together, these observations suggest that synapse subtypes with rapid proteostasis play a vital role in the brain's adaptive responses to SD and genetic perturbations.

CellPress
OPEN ACCESS

Implications for cognition

Our current understanding of the mechanisms behind the impairments in memory consolidation induced by SD is limited and generally centered around models of synaptic plasticity. Our study suggests that the synaptome and SA may play a role from several perspectives. First, we found that SD specifically affects the SA of the HPF and cortex, regions crucial for memory consolidation during sleep.[19,20] Second, as a consequence of the altered SA, the electrophysiological responses in the CA1sr to theta burst and gamma train patterns were modified. These changes could impact the encoding and learning phases in the active state of the animal, ultimately affecting memory consolidation during sleep.[21,22] Furthermore, modifications in sharp-wave ripple activity could contribute further to memory impairments associated with SD by influencing replay activity supported by these oscillations during periods of quiet waking (when sleep-like patterns are observed in the HPF).[23,24] Superimposed on these electrophysiological functions is the role of protein turnover, which Francis Crick highlighted almost four decades ago[25] as a crucial determinant of memory consolidation. The imbalance between synapses with long and short protein lifetimes caused by SD might itself interfere with consolidation and even synergize with the altered electrophysiological responses.

Sleep disruption and psychiatric disorders

Modulation of the SA offers a novel nexus between sleep disruption and psychiatric disorders. Schizophrenia is a polygenic disorder that targets the synapse proteome,[26–31] and similar to our results with SD, animal models of schizophrenia show altered SA of PSD95-expressing synapses and alterations in the responses of the SA to patterns of neural activity.[10] This raises the possibility that individuals with schizophrenia could be more severely impacted by sleep disruption, consistent with reports that SD contributes to their delusions and hallucinations.[32–34] Moreover, acute sleep disruption and extended wakefulness alter mood and affect and motoric, social, and sexual behaviors that are phenotypes associated with mania and depression and linked to synapses.[35] Thus, the altered SA in individuals arising from a genetic susceptibility to psychiatric disorders may render them vulnerable to the deleterious effects of sleep and circadian disruption.

Future studies of the synaptome architecture in sleep and circadian biology

When focused on the hippocampus, studies using SD have shown either a decrease or increase in spine numbers and contacts, a discrepancy that can be explained by the methods used to induce SD and the age of the animal used.[5] When using gentle handling, as we do here, results point toward a reduction in spine structure, aligning with our current findings.[5] These observations, added to the findings showing an impairment in mechanisms of hippocampal translation regulation,[36] long-term potentiation of synaptic transmission,[18] and spine physiology (e.g., cofilin),[37] provide strong support for the hypothesis that extended wakefulness is detrimental to the hippocampal physiology underlying memory formation.

This study was limited to the use of two postsynaptic scaffold proteins, which are major structural proteins in excitatory synapses. Proteomic studies have revealed many synaptic proteins that are modulated during sleep, and judicious choice of these markers for synaptome mapping will likely uncover dynamic properties of the SA during sleep states and with different durations of wakefulness.

Synaptome mapping is a powerful tool that has wide applications in sleep and circadian research, including examination of the role of REM sleep, chronic sleep deprivation, sleep recovery, and the role of extended wakefulness. These studies can be combined with genetic models of disease to explore how sleep modifies the SA of the brain in these disorders. The concept of the synaptome and SA of the brain offers a new framework for understanding the synaptic basis of SD and suggests that interventions targeting specific subtypes of synapses could potentially prevent or reverse cognitive impairments associated with SD, aging, and psychiatric disorders.

STAR★METHODS

Detailed methods are provided in the online version of this paper and include the following:

- KEY RESOURCES TABLE
- RESOURCE AVAILABILITY
  - Lead contact
  - Materials availability
  - Data and code availability
- EXPERIMENTAL MODEL AND SUBJECT DETAILS
  - Animals
- METHOD DETAILS
  - Housing conditions
  - Circadian sleep-wake cycle study
  - Sleep deprivation study
  - Tissue collection and sectioning
  - Tissue preparation
  - Spinning-disk confocal microscopy
- QUANTIFICATION AND STATISTICAL ANALYSIS
  - Synaptome mapping pipeline
  - Classification of synapse types and subtypes
  - Calculation of the long and short protein-turnover rates of synapse subtypes
  - Cohen's d formula
  - Bayesian analysis
  - Computational modeling of synaptic responses
  - Synaptic scaling representing timepoint and SD state
  - Computational model of spatial differences in PSD95 and SAP102
  - Estimation of free model parameters from data
  - Synaptic scaling representing timepoint and SD state

SUPPLEMENTAL INFORMATION

Supplemental information can be found online at https://doi.org/10.1016/j.cub.2024.07.032.

ACKNOWLEDGMENTS

We would like to thank B. Notman, T. Wong, and E. Sigfridsson for laboratory support; B. Koniaris for assistance in computational analysis; C. Davey for editing; and D. Maizels for artwork. Funding was provided by the European Research Council (ERC) under the European Union's Horizon 2020 Research and Innovation Programme (695568 SYNNOVATE) to D.K., E.B., Z.Q., and S.G.N.G.; Wellcome Technology Development grant (202932/Z/16/Z) to R.G., Z.Q., and S.G.N.G.; the Simons Foundation Autism Research Initiative (529085) to N.H.K. and S.G.N.G.; the Wellcome Trust (209099/Z/17/Z) to J.S.; and the Leverhulme Trust (RPG-2020-340) to J.S. D.K. was funded in

part by a Principal's Career Development PhD Scholarship from the University of Edinburgh.

For the purpose of open access, the author has applied a CC-BY public copyright license to any author accepted manuscript version arising from this submission.

AUTHOR CONTRIBUTIONS

D.K. planned the experiments; collected, imaged, and analyzed brain samples; and performed image and data analyses. Z.Q. developed software and performed image and data analyses. E.F. analyzed data and performed computational modeling of neural activity. R.G. performed data handling and generated brain maps. E.B. provided data on synapse protein turnover. N.H.K. and J.S. provided supervision. S.G.N.G. supervised the project and wrote the manuscript.

DECLARATION OF INTERESTS

The authors declare no competing interests.

Received: December 11, 2023
Revised: May 17, 2024
Accepted: July 5, 2024
Published: August 2, 2024

REFERENCES

1. Chattu, V.K., Manzar, M.D., Kumary, S., Burman, D., Spence, D.W., and Pandi-Perumal, S.R. (2018). The Global Problem of Insufficient Sleep and Its Serious Public Health Implications. Healthcare (Basel) *7*, 1.
2. Brodt, S., Inostroza, M., Niethard, N., and Born, J. (2023). Sleep—A brain-state serving systems memory consolidation. Neuron *111*, 1050–1075.
3. Bayes, A., van de Lagemaat, L.N., Collins, M.O., Croning, M.D., Whittle, I.R., Choudhary, J.S., and Grant, S.G. (2011). Characterization of the proteome, diseases and evolution of the human postsynaptic density. Nat. Neurosci. *14*, 19–21.
4. Cirelli, C., and Tononi, G. (2020). Effects of sleep and waking on the synaptic ultrastructure. Philos. Trans. R. Soc. Lond. B Biol. Sci. *375*, 20190235.
5. Havekes, R., and Aton, S.J. (2020). Impacts of Sleep Loss versus Waking Experience on Brain Plasticity: Parallel or Orthogonal? Trends Neurosci. *43*, 385–393.
6. Puentes-Mestril, C., and Aton, S.J. (2017). Linking Network Activity to Synaptic Plasticity during Sleep: Hypotheses and Recent Data. Front. Neural Circuits *11*, 61.
7. Raven, F., Van der Zee, E.A., Meerlo, P., and Havekes, R. (2018). The role of sleep in regulating structural plasticity and synaptic strength: implications for memory and cognitive function. Sleep Med. Rev. *39*, 3–11.
8. Cizeron, M., Qiu, Z., Koniaris, B., Gokhale, R., Komiyama, N.H., Fransen, E., and Grant, S.G.N. (2020). A brain-wide atlas of synapses across the mouse lifespan. Science *369*, 270–275.
9. Tomas-Roca, L., Qiu, Z., Fransen, E., Gokhale, R., Bulovaite, E., Price, D.J., Komiyama, N.H., and Grant, S.G.N. (2022). Developmental disruption and restoration of brain synaptome architecture in the murine Pax6 neurodevelopmental disease model. Nat. Commun. *13*, 6836.
10. Zhu, F., Cizeron, M., Qiu, Z., Benavides-Piccione, R., Kopanitsa, M.V., Skene, N.G., Koniaris, B., DeFelipe, J., Fransen, E., Komiyama, N.H., and Grant, S.G.N. (2018). Architecture of the Mouse Brain Synaptome. Neuron *99*, 781–799.e10.
11. Bulovaite, E., Qiu, Z., Kratschke, M., Zgraj, A., Fricker, D.G., Tuck, E.J., Gokhale, R., Koniaris, B., Jami, S.A., Merino-Serrais, P., et al. (2022). A brain atlas of synapse protein lifetime across the mouse lifespan. Neuron *110*, 4057–4073.e8.
12. Grant, S.G.N. (2018). The Synaptomic Theory of Behavior and Brain Disease. Cold Spring Harb. Symp. Quant. Biol. *83*, 45–56.
13. Frank, R.A., Komiyama, N.H., Ryan, T.J., Zhu, F., O'Dell, T.J., and Grant, S.G. (2016). NMDA receptors are selectively partitioned into complexes and supercomplexes during synapse maturation. Nat. Commun. *7*, 11264.
14. Carlisle, H.J., Fink, A.E., Grant, S.G., and O'Dell, T.J. (2008). Opposing effects of PSD-93 and PSD-95 on long-term potentiation and spike timing-dependent plasticity. J. Physiol. *586*, 5885–5900.
15. Migaud, M., Charlesworth, P., Dempster, M., Webster, L.C., Watabe, A.M., Makhinson, M., He, Y., Ramsay, M.F., Morris, R.G., Morrison, J.H., et al. (1998). Enhanced long-term potentiation and impaired learning in mice with mutant postsynaptic density-95 protein. Nature *396*, 433–439.
16. Nithianantharajah, J., Komiyama, N.H., McKechanie, A., Johnstone, M., Blackwood, D.H., St Clair, D., Emes, R.D., van de Lagemaat, L.N., Saksida, L.M., Bussey, T.J., and Grant, S.G.N. (2013). Synaptic scaffold evolution generated components of vertebrate cognitive complexity. Nat. Neurosci. *16*, 16–24.
17. Lyons, L.C., Chatterjee, S., Vanrobaeys, Y., Gaine, M.E., and Abel, T. (2020). Translational changes induced by acute sleep deprivation uncovered by TRAP-Seq. Mol. Brain *13*, 165.
18. Vecsey, C.G., Baillie, G.S., Jaganath, D., Havekes, R., Daniels, A., Wimmer, M., Huang, T., Brown, K.M., Li, X.Y., Descalzi, G., et al. (2009). Sleep deprivation impairs cAMP signalling in the hippocampus. Nature *461*, 1122–1125.
19. Marshall, L., and Born, J. (2007). The contribution of sleep to hippocampus-dependent memory consolidation. Trends Cogn. Sci. *11*, 442–450.
20. Ognjanovski, N., Maruyama, D., Lashner, N., Zochowski, M., and Aton, S.J. (2014). CA1 hippocampal network activity changes during sleep-dependent memory consolidation. Front. Syst. Neurosci. *8*, 61.
21. Cantero, J.L., and Atienza, M. (2005). The role of neural synchronization in the emergence of cognition across the wake-sleep cycle. Rev. Neurosci. *16*, 69–83.
22. Ferrara, M., Moroni, F., De Gennaro, L., and Nobili, L. (2012). Hippocampal sleep features: relations to human memory function. Front. Neurol. *3*, 57.
23. Yamamoto, J., and Tonegawa, S. (2017). Direct Medial Entorhinal Cortex Input to Hippocampal CA1 Is Crucial for Extended Quiet Awake Replay. Neuron *96*, 217–227.e4.
24. Joo, H.R., and Frank, L.M. (2018). The hippocampal sharp wave-ripple in memory retrieval for immediate use and consolidation. Nat. Rev. Neurosci. *19*, 744–757.
25. Crick, F. (1984). Neurobiology: Memory and molecular turnover. Nature *312*, 101.
26. Trubetskoy, V., Pardinas, A.F., Qi, T., Panagiotaropoulou, G., Awasthi, S., Bigdeli, T.B., Bryois, J., Chen, C.Y., Dennison, C.A., Hall, L.S., et al. (2022). Mapping genomic loci implicates genes and synaptic biology in schizophrenia. Nature *604*, 502–508.
27. Skene, N.G., Roy, M., and Grant, S.G. (2017). A genomic lifespan program that reorganises the young adult brain is targeted in schizophrenia. Elife *6*, e17915.
28. Singh, T., Poterba, T., Curtis, D., Akil, H., Al Eissa, M., Barchas, J.D., Bass, N., Bigdeli, T.B., Breen, G., Bromet, E.J., et al. (2022). Rare coding variants in ten genes confer substantial risk for schizophrenia. Nature *604*, 509–516.
29. Kirov, G., Pocklington, A.J., Holmans, P., Ivanov, D., Ikeda, M., Ruderfer, D., Moran, J., Chambert, K., Toncheva, D., Georgieva, L., et al. (2012). De novo CNV analysis implicates specific abnormalities of postsynaptic signalling complexes in the pathogenesis of schizophrenia. Mol. Psychiatry *17*, 142–153.
30. Fromer, M., Pocklington, A.J., Kavanagh, D.H., Williams, H.J., Dwyer, S., Gormley, P., Georgieva, L., Rees, E., Palta, P., Ruderfer, D.M., et al. (2014). De novo mutations in schizophrenia implicate synaptic networks. Nature *506*, 179–184.
31. Fernandez, E., Collins, M.O., Uren, R.T., Kopanitsa, M.V., Komiyama, N.H., Croning, M.D., Zografos, L., Armstrong, J.D., Choudhary, J.S., and Grant, S.G. (2009). Targeted tandem affinity purification of PSD-95

CellPress
OPEN ACCESS

recovers core postsynaptic complexes and schizophrenia susceptibility proteins. Mol. Syst. Biol. *5*, 269.

32. Wulff, K., Dijk, D.J., Middleton, B., Foster, R.G., and Joyce, E.M. (2012). Sleep and circadian rhythm disruption in schizophrenia. Br. J. Psychiatry *200*, 308–316.
33. Reeve, S., Sheaves, B., and Freeman, D. (2015). The role of sleep dysfunction in the occurrence of delusions and hallucinations: A systematic review. Clin. Psychol. Rev. *42*, 96–115.
34. Freeman, D., Sheaves, B., Waite, F., Harvey, A.G., and Harrison, P.J. (2020). Sleep disturbance and psychiatric disorders. Lancet Psychiatr. *7*, 628–637.
35. Wu, M., Zhang, X., Feng, S., Freda, S.N., Kumari, P., Dumrongprechachan, V., and Kozorovitskiy, Y. (2024). Dopamine pathways mediating affective state transitions after sleep loss. Neuron *112*, 141–154.e8.
36. Tudor, J.C., Davis, E.J., Peixoto, L., Wimmer, M.E., van Tilborg, E., Park, A.J., Poplawski, S.G., Chung, C.W., Havekes, R., Huang, J., et al. (2016). Sleep deprivation impairs memory by attenuating mTORC1-dependent protein synthesis. Sci. Signal. *9*, ra41.
37. Havekes, R., Park, A.J., Tudor, J.C., Luczak, V.G., Hansen, R.T., Ferri, S.L., Bruinenberg, V.M., Poplawski, S.G., Day, J.P., Aton, S.J., et al. (2016). Sleep deprivation causes memory deficits by negatively impacting neuronal connectivity in hippocampal area CA1. Elife *5*, e13424.
38. Cohen, J. (2013). Statistical power analysis for the behavioral sciences (Academic press).
39. Kruschke, J.K. (2013). Bayesian estimation supersedes the t test. J. Exp. Psychol. Gen. *142*, 573–603.
40. Horner, A.E., McLaughlin, C.L., Afinowi, N.O., Bussey, T.J., Saksida, L.M., Komiyama, N.H., Grant, S.G., and Kopanitsa, M.V. (2018). Enhanced cognition and dysregulated hippocampal synaptic physiology in mice with a heterozygous deletion of PSD-95. Eur. J. Neurosci. *47*, 164–176.
41. Kopanitsa, M.V., van de Lagemaat, L.N., Afinowi, N.O., Strathdee, D.J., Strathdee, K.E., Fricker, D.G., Tuck, E.J., Elsegood, K.A., Croning, M.D., Komiyama, N.H., et al. (2018). A combinatorial postsynaptic molecular mechanism converts patterns of nerve impulses into the behavioral repertoire. Preprint at bioRxiv. https://doi.org/10.1101/500447.
42. Levy, J.M., Chen, X., Reese, T.S., and Nicoll, R.A. (2015). Synaptic consolidation normalizes AMPAR quantal size following MAGUK loss. Neuron *87*, 534–548.
43. Tsodyks, M.V., and Markram, H. (1997). The neural code between neocortical pyramidal neurons depends on neurotransmitter release probability. Proc. Natl. Acad. Sci. USA *94*, 719–723.
44. Varela, J.A., Sen, K., Gibson, J., Fost, J., Abbott, L.F., and Nelson, S.B. (1997). A quantitative description of short-term plasticity at excitatory synapses in layer 2/3 of rat primary visual cortex. J. Neurosci. *17*, 7926–7940.
45. Zucker, R.S., and Regehr, W.G. (2002). Short-term synaptic plasticity. Annu. Rev. Physiol. *64*, 355–405.

STAR★METHODS

KEY RESOURCES TABLE

REAGENT or RESOURCE	SOURCE	IDENTIFIER
Chemicals, peptides, and recombinant proteins		
Pentobarbital (Euthatal)	Merial Animal Health	N/A
Phosphate-Buffered Saline (PBS)	Oxoid, Basingstoke, UK	Cat#10209252
Paraformaldehyde (PFA)	Alfa Aesar, Heysham, UK	Cat#A11313
Sucrose	VWR Chemicals, Lutterworth, UK	Cat#470302-808
Optimal Cutting Temperature medium (OCT)	VWR International	Cat#361603E
Isopentane	Sigma-Aldrich	Cat#59060
Glycerol (BioXtra >99%)	Sigma-Aldrich	Cat#G6279
Mowiol	Calbiochem	Cat#47-590-4100GM
1,4-diazabicyclo[2.2.2]octane (DABCO)	Sigma-Aldrich	Cat#D27802
Deposited data		
Datasets	This paper	https://doi.org/10.7488/ds/7779
Experimental models: Organisms/strains		
Mouse: C57BL/6 J, *Psd95*$^{eGFP/eGFP}$; *Sap102*$^{mKO2/mKO2}$ knock-in	Zhu et al.[10]	N/A
Software and algorithms		
Andor iQ2	Oxford Instruments Andor	https://andor.oxinst.cn/products/iq-live-cell-imaging-software/andor-iq3
MATLAB	Mathworks	https://www.mathworks.com/products/matlab
ImageJ	Fiji	https://fiji.sc/
Python	Python Software Foundation	https://www.python.org/
GraphPad Prism	GraphPad Software	https://www.graphpad.com/
Allen Reference Atlas	Allen Institute	http://mouse.brain-map.org/
Other		
Plastic cubic molds	Sigma-Aldrich	Cat#E6032
SuperFrost Plus microscopy slides	Thermo Scientific	Cat#1178T42
Round glass coverslip (1.5mm)	VWR International	Cat#10026-136

RESOURCE AVAILABILITY

Lead contact

Further information and requests for resources and reagents should be directed to and will be fulfilled by the lead contact, Seth G. N. Grant (seth.grant@ed.ac.uk).

Materials availability

This study did not generate new unique reagents.

Data and code availability

All data have been deposited on the Edinburgh Datashare website and are publicly available as of the date of publication. DOIs are listed in the key resources table. Any additional information required to reanalyse the data reported in this paper is available from the lead contact upon request.

EXPERIMENTAL MODEL AND SUBJECT DETAILS

Animals

Animal procedures were performed in accordance with UK Home Office regulations and approved by Edinburgh University Director of Biological Services. Generation of the *Psd95*$^{eGFP/eGFP}$;*Sap102*$^{mKO2/mKO2}$ knock-in mouse line using C57BL/6 J mice and its

characterisation have been described.[10] The same study[10] was used to estimate the sample size needed, leveraging a t-test analysis. Adult 3-month-old mice were used for this study.

METHOD DETAILS

Housing conditions

Mice were transferred from their home cage to a new, conventional, enrichment-free cage, where they were single-housed for a 3-day habituation period (days 0–2) and for the day of the experiment (day 3). The animals had *ad libitum* access to water and food and were kept in a quiet room of ~22°C and 12-h light:dark cycle (lights on 7 a.m., lights off 7 p.m.). For the purposes of masking the experimental groups to the experimenter during processing of samples and analysis, each animal received a randomized ID number which was maintained throughout tissue processing and imaging.

Circadian sleep-wake cycle study

A group of 30 animals was split into three equivalent groups of 10 animals, each comprising 5 males and 5 females. The mice were undisturbed during days 0–2 and their brain tissue was collected at ZT6, ZT11 or ZT23 on day 3 based on their assigned group.

Sleep deprivation study

A group of 18 animals was split into two groups: the ZT6 group (5 males and 3 females) and the ZT6SD group (6 males and 4 females). All mice were handled daily for 10 min by the experimenter between 07:30 a.m. and 08:00 a.m. during habituation days 0–2. On day 3, the mice were either left undisturbed (ZT6 group) or were kept awake by gentle handling (ZT6SD group), which comprised gentle auditory and tactile stimulation (tapping on the cage and touching the animals with a brush) when the animals were visually observed to have fallen asleep. The brain tissue of animals of both groups was collected 6 h after lights-on (time point ZT6). The experimenter was present in the room during the 6 h of the experiment on day 3 for both groups.

Tissue collection and sectioning

At the collection time points mice were anaesthetised by intraperitoneal injection of a lethal dose of 0.1 mL 20% pentobarbital (Euthatal, Merial Animal Health). After complete anesthesia, 10 mL phosphate-buffered saline (PBS; Oxoid, Basingstoke, UK) were used per animal for cardiac perfusion, followed by 10 mL 4% (v/v) paraformaldehyde (PFA; Alfa Aesar, Heysham, UK) for fixation, both solutions at 4°C. Whole brains were dissected out and immediately postfixed at 4°C in 5 mL 4% PFA for 4 h, before being transferred to 5 mL 30% (w/v) sucrose (VWR Chemicals, Lutterworth, UK) in 1×PBS at 4°C for ~72 h. In preparation for embedding, brains were kept for 1 h in a 1:1 solution of 30% sucrose and Optimal Cutting Temperature medium (OCT, VWR International) at 4°C. Finally, brains were placed in plastic cubic molds (Sigma-Aldrich) containing OCT for embedding, and were immediately frozen in a container with ~10 mL isopentane (Sigma-Aldrich) placed in liquid nitrogen. After completion of embedding, the tissue was stored at −80°C. Each brain was sectioned in the coronal −1.9 mm bregma level at 18 μm thickness using a cryostat (NX70, Thermo Fisher Scientific, Gloucester, UK), and placed on glass SuperFrost Plus microscopy slides (Thermo Scientific).

Tissue preparation

In preparation for imaging, the brain tissue slices were rinsed with ice-cold 100 μL PBS for 5 min. Excess PBS was then removed with a Kimwipe tissue, and 12 μL Mowiol mounting medium (96 g glycerol (Sigma-Aldrich, BioXtra >99%), 38.4 g Mowiol (Calbiochem), 192 mL 0.2 M Tris buffer (pH 8.5), 96 mL milliQ water (18.2 MΩ)) with 2.5% DABCO (1,4-diazabicyclo[2.2.2]octane; Sigma-Aldrich) was added to each section for optimal imaging without absorption, autofluorescence or light scattering, and for the prevention of photobleaching. Finally, each slice was covered with a 13 mm diameter, 1.5 mm thick round glass coverslip (VWR International) and imaged the following day.

Spinning-disk confocal microscopy

Image capture employed an Andor Revolution XDi spinning-disk microscopy system equipped with a Yokogawa CSU-X1 50 μm pinhole spinning disk, an Olympus uPlanSAPO 100X oil-immersion lens (NA 1.4), and an Andor iXon Ultra monochrome back-illuminated EMCCD camera capturing images with 16-bit depth and 512 × 512 pixels (pixel size of 0.084 μm). Frame averaging of 2 and 250 EMCCD gain was applied to all synaptome mapping scans. The imaging settings for each fluorophore-tagged protein in the synaptome mapping experiments were: PSD95eGFP 488 nm, QUAD emission filter, exposure time 0.07 s, power 20%; and for SAP102mKO2 561 nm, QUAD emission filter, exposure time 0.1 s, power 40%. A single mosaic grid was used to cover each entire brain section with an adaptive z-focus set up by the user to follow the unevenness of the tissue using Andor iQ2 software.

QUANTIFICATION AND STATISTICAL ANALYSIS

Synaptome mapping pipeline

The synaptome mapping (SynMap) technique[10] was established and standardized to systematically map individual PSD95eGFP puncta across the entire brain. Using deep learning methods developed in house, SynMap comprises a sequence of automated image analysis procedures that includes puncta detection, colocalization, classification, and map reconstruction, among others. To

define the anatomical regions within SynMap, manual delineation was employed, using the Allen Reference Atlas (http://mouse.brain-map.org/) as a guiding resource for identifying the boundaries of distinct anatomical areas.

Classification of synapse types and subtypes

Advanced machine learning approaches[10] were developed to detect and segment PSD95eGFP and SAP102mKO2 puncta from SDM images. Puncta intensity, size and shape parameters were then quantified for each punctum at the single-synapse scale. Using advanced unsupervised machine learning developed in house,[10] their parameters were used as input to build a hierarchical catalogue comprising of: firstly 3 main types, synapse expressing only PSD95 (PSD95-only), expressing only SAP102 (SAP102-only) and both (Colocalized); and then further 37 subtypes. Details can be referred to our previous papers.[8,10]

Synapse diversity ranges from 0 to 1 and quantifies the density difference between different subtypes: a large diversity indicates all subtypes have similar densities. A detailed description and formal mathematical definition is provided in the methods section of Zhu et al.[10]

Calculation of the long and short protein-turnover rates of synapse subtypes

In our previous study,[11] we quantified the lifetime of PSD95 in 30 synapse subtypes that express PSD95. was then measured by visualizing the duration of the labeled protein. The top 6 subtypes with longest turnover rate were considered as LPL and the bottom 6 were SPL synapses.

Cohen's d formula

Cohen's d effect size was calculated as per Cohen[38] as follows:

$$d = \frac{\underline{x}_1 - \underline{x}_2}{s}$$

where $\underline{x}$ is the mean of one of the groups, where s is the pooled standard deviation as

$$s = \sqrt{\frac{(n_1 - 1)s_1^2 + (n_2 - 1)s_2^2}{n_1 + n_2 - 2}}$$

where the variance (s^2) of one of the groups as

$$s_1^2 = \frac{1}{n_1 - 1}\sum_{i=1}^{n_1}\left(x_{1,i} - \underline{x}_1\right)^2$$

Bayesian analysis

Bayesian estimation[39] as used previously was applied to provide a more objective statistical test of the estimate the significance of the effects of SD on synaptome maps, including basic PSD95 parameters, subtype density and diversity.

A two-stage Bayesian estimation approach was used to examine the differential effects of SD on SPL and LPL subtypes. In the first stage, a probability distribution of effect size for both SPL and LPL regarding SD was generated. In the second stage, an additional Bayesian estimation was applied to the previously estimated distribution to determine the effects of SD on SPL or LPL subtypes. This two-stage testing method was implemented for each subregion to generate significant p-values and median Cohen's d values. Lastly, Benjamini-Hochberg corrections were applied to compute the corrected p-values across all brain regions.

Computational modeling of synaptic responses

Computational modeling of synaptic temporal responses was based on our previously described model[8–10] representing physiology in the hippocampus, briefly outlined below. Here we modified this model to include the effects of timepoint and SD state. These synaptic models simulate changes in synapse temporal responses, EPSP amplitudes, short-term plasticity and temporal summation based on observations from neurons where PSD95 and SAP102 expression is altered.[14,15,40–42] For a description of the modeling of how the spatial differences in PSD95 and SAP102 affects individual synaptic time dynamics, see the section "Computational Model of Spatial Differences in PSD95 and SAP102" below.

Synaptic scaling representing timepoint and SD state

As described in previous work,[8,10] the size of PSD95 and SAP102 synapses along the radial and tangential directions of the hippocampus (Figures 1F and 3) is derived from the fluorescence intensity measurement of individual synaptic puncta and represented by color intensity; PSD95 (green) and SAP102 (magenta). These size values were used to scale the synaptic properties of the computational model to represent differences in animal group (timepoint and SD state). To model synaptic physiology corresponding to 3 month old animals in control and sleep deprived animals, differences in the size of PSD95 and SAP102 along radial as well as tangential directions of hippocampus were computed as outlined below.

The hippocampus was delineated into four tangential subregions (CA1, CA2, CA3 and dentate gyrus) and four radial layers (for CA1-3 stratum lacunosum-moleculare, stratum radiatum, stratum pyramidale and stratum oriens and for dentate gyrus the superior

molecular layer, polymorphic layer, inferior granular layer and inferior molecular layer), for each of the animal groups. Next, we computed the geometric mean over individuals (N = 8 for ZT6 and N = 10 for the other groups) of PSD95 and SAP102 puncta size. Then, for each protein, we normalized data according to the following. We computed the directional gradient (largest minus smallest value) along each direction (radial or tangential) for each animal group and from this identified the minimum and the maximum span over all animal groups. Normalization was done by subtracting the minimum span and dividing by that maximum span. Thus, for radial and tangential size values of PSD95 and SAP102 puncta, the expression was compared over all animal groups. This relative size was used to scale the spatial distribution of synaptic values used in previous work.[10] This scaling thus allows for a comparison of animals at different daily time points and SD.

Computational model of spatial differences in PSD95 and SAP102

The following text, adapted from Zhu et al.[10] models data at 3 months age.

Synaptic responses

Synaptic EPSPs were described by a bi-exponential function. Parameters $\tau1$ and $\tau2$ were set to reproduce a fast ionotropic synaptic AMPA-type time course.

$$V = A_e \times (exp(-t/\tau1) - exp(-t/\tau2))$$

where $A_e = \Pi_i 1 \times A_{tfi} \times A_{tdi}$, $\tau1$ = 3.0 ms, $\tau2$ = 0.4 ms, i index of all preceding spikes.

Short-term synaptic changes followed a formalism described by Tsodyks and Markram[43] and Varela et al.[44] We included one fast and one slow facilitating component and one depressing component, all which affected synaptic responses following the triggering one. In all figures, amplitudes were shown normalized to the amplitude of the first response.

Depression model

$$Adi = Ad \times exp(-\Delta t/\tau d)$$

where

Δt_i is the time between the preceding event i and the present event.

$A_d = A_{d0} \times S_{Ad}$, S_{Ad} is normalized tangential PSD95 size factor*, [0, 1].

$\tau_d = \tau_{d0} \times S_{Td}$, S_{Td} is normalized radial PSD95 size factor*, [0, 1].

$A_{tdi} = \max(\Sigma_i(1-A_{di}), 0)$, total depressing response was limited to positive values.

Fast facilitation, F1

$$A_{fi} = A_f \times exp(-\Delta t_i/\tau_f)$$

where

$A_f = A_{f0} \times S_{Af}$, S_{Af} is normalized tangential SAP102 size factor*, [0, 1].

$\tau_f = \tau_{f0} \times S_{Tf}$, S_{Tf} is normalized radial SAP102 size factor*, [0, 1].

Slow facilitation, F2

$$A_{si} = A_s \times exp(-\Delta t_i/\tau_s)$$

where

$A_s = A_{s0}$

$\tau_s = \tau_{s0}$

The total facilitatory response had a saturation at 3.3 times the unit response.[45]

$$A_{tfi} = \min(1 + \Sigma_i(A_{fi} + A_{si}), 3.3)$$

*The experimental tangential and radial profile data of PSD95 and SAP102 normalized size data[10] were used to set the differential model parameter values along the spatial dimension.

Estimation of free model parameters from data

Free model parameters were set to replicate experimental data of synaptic amplitudes in response to a 10 cycle theta-burst protocol.[41] The model was fitted to amplitude data from theta-burst experiments for bursts 1, 2, 8 and 10 in a 10 burst protocol. Verification tests showed that including a second, potentially slower, depression factor did not significantly reduce the fitting error. Furthermore, PSD95-related parameters were set to replicate the respective paired-pulse facilitation fractional differences between recordings in tissues from WT and knockout animals (IPI = 25, 50, 100, 200 ms).[14] For the estimation of the parameters in knockout models, only Ad0, τd0 and Af0 were allowed to change. Verification and parameter sensitivity tests showed that inclusion of the three other parameters did not significantly affect the fitting error. Simulations were performed using MATLAB, R2022b with a time discretization of 1 ms. Time constants τ in unit ms and amplitudes in a.u (arbitrary units).

Synaptic scaling representing timepoint and SD state

The size of PSD95 and SAP102 synapses along the radial and tangential directions of the hippocampus (Figure 3) is derived from the fluorescence intensity measurement of individual synaptic puncta and represented by color intensity, also described in previous work[8,10]; PSD95 (green) and SAP102 (magenta). These size values were used to scale the synaptic properties of the computational model to represent differences in animal group (timepoint and SD state). To model synaptic physiology corresponding to 3-month-old animals in control and sleep deprived animals, differences in the size of PSD95 and SAP102 along radial as well as tangential directions of hippocampus were computed as follows.

(1) The hippocampus was delineated into four tangential subregions (CA1, CA2, CA3 and dentate gyrus) and four radial layers (for CA1-3 stratum lacunosum-moleculare, stratum radiatum, stratum pyramidale and stratum oriens and for dentate gyrus the superior molecular layer, polymorphic layer, inferior granular layer and inferior molecular layer), for each of the animal groups.
(2) Next, we computed the geometric mean over individuals (N = 8 for ZT6 and N = 10 for the other groups) of PSD95 and SAP102 puncta size.
(3) Then, for each protein, we normalized data according to the following. We computed the directional gradient (largest minus smallest value) along each direction (radial or tangential) for each animal group and from this identified the minimum and the maximum span over all animal groups. Normalization was done by subtracting the minimum span and dividing by that maximum span. Thus, for radial and tangential size values of PSD95 and SAP102 puncta, the expression was compared over all animal groups. This relative size was used to scale the spatial distribution of synaptic values used in previous work.[10] This scaling thus allows for a comparison of animals at different daily time points and SD.

Current Biology, Volume *34*

Supplemental Information

Sleep maintains excitatory synapse

diversity in the cortex and hippocampus

Dimitra Koukaroudi, Zhen Qiu, Erik Fransén, Ragini Gokhale, Edita Bulovaite, Noboru H. Komiyama, Julie Seibt, and Seth G.N. Grant

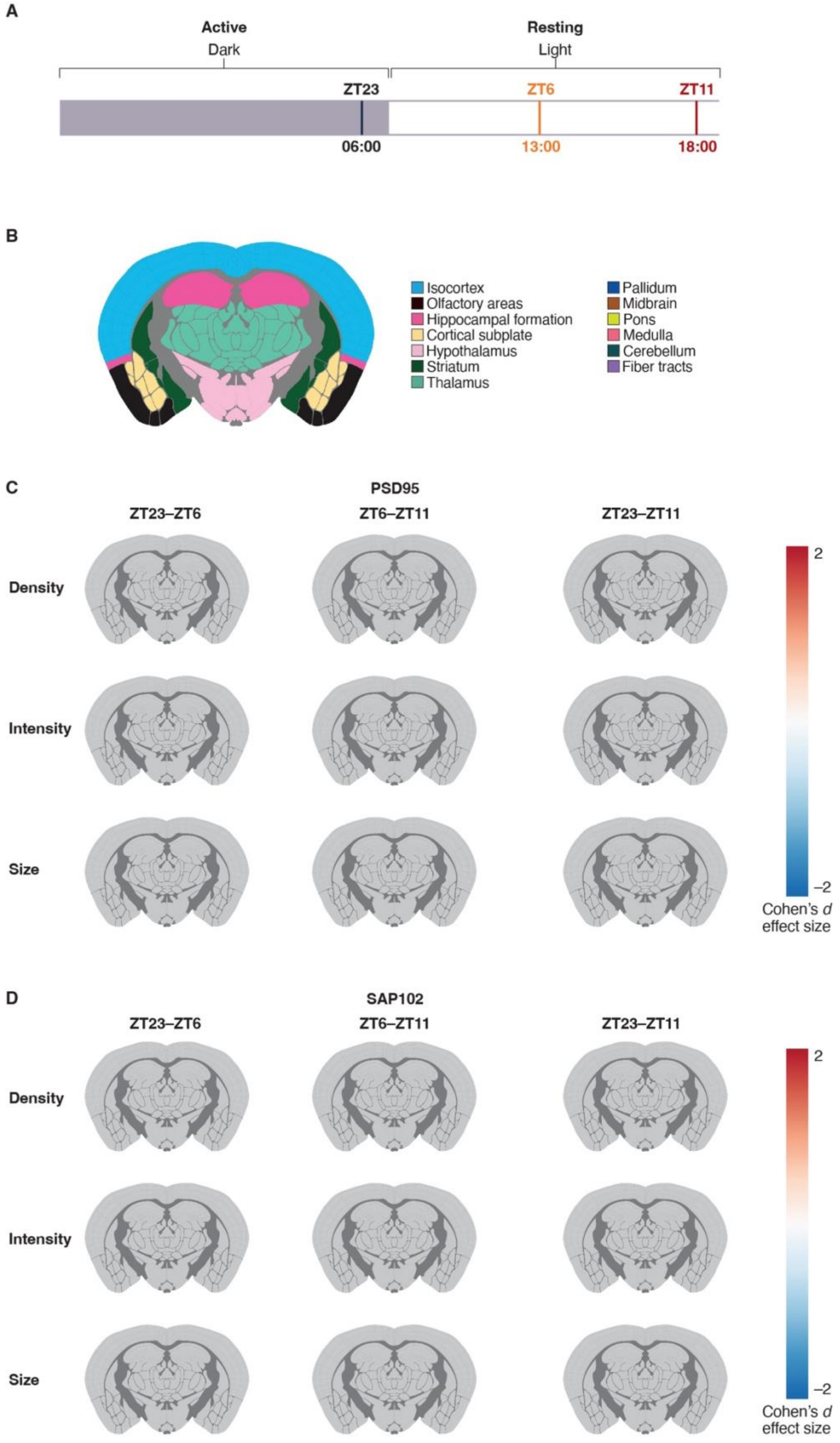
A
Active
Dark
Resting
Light
ZT23
ZT6
ZT11
06:00
13:00
18:00
B
Isocortex
Olfactory areas
Hippocampal formation
Cortical subplate
Hypothalamus
Striatum
Thalamus
Pallidum
Midbrain
Pons
Medulla
Cerebellum
Fiber tracts
C
PSD95
ZT23–ZT6
ZT6–ZT11
ZT23–ZT11
Density
Intensity
Size
2
−2
Cohen's d effect size
D
SAP102
ZT23–ZT6
ZT6–ZT11
ZT23–ZT11
Density
Intensity
Size
2
−2
Cohen's d effect size

Figure S1 The synaptome architecture of PSD95 and SAP102 puncta does not change in the circadian cycle. Related to Figure 2.

(A) Schematic representation of light/dark cycle, with zeitgeiber time (ZT) showing when mice were sampled (ZT23, ZT6, ZT11) and corresponding 24-hour clock (06:00, 13:00, 18:00). (B) Key for brain regions shown in maps (C,D). (C)Synaptome maps of PSD95 puncta density (top row), intensity (middle row), size (bottom row) changes for periods ZT23-ZT6 (left column), ZT6-ZT11 (middle column), ZT23-ZT11 (right column) in brain regions shows no significant changes (grey; P>0.05, Bayesian test with Benjamini-Hochberg correction). (D) Synaptome maps of SAP102 puncta density (top row), intensity (middle row), size (bottom row) changes for periods ZT23-ZT6 (left column), ZT6-ZT11 (middle column), ZT23-ZT11 (right column) in brain regions shows no significant changes (grey; P>0.05, Bayesian test with Benjamini-Hochberg correction).

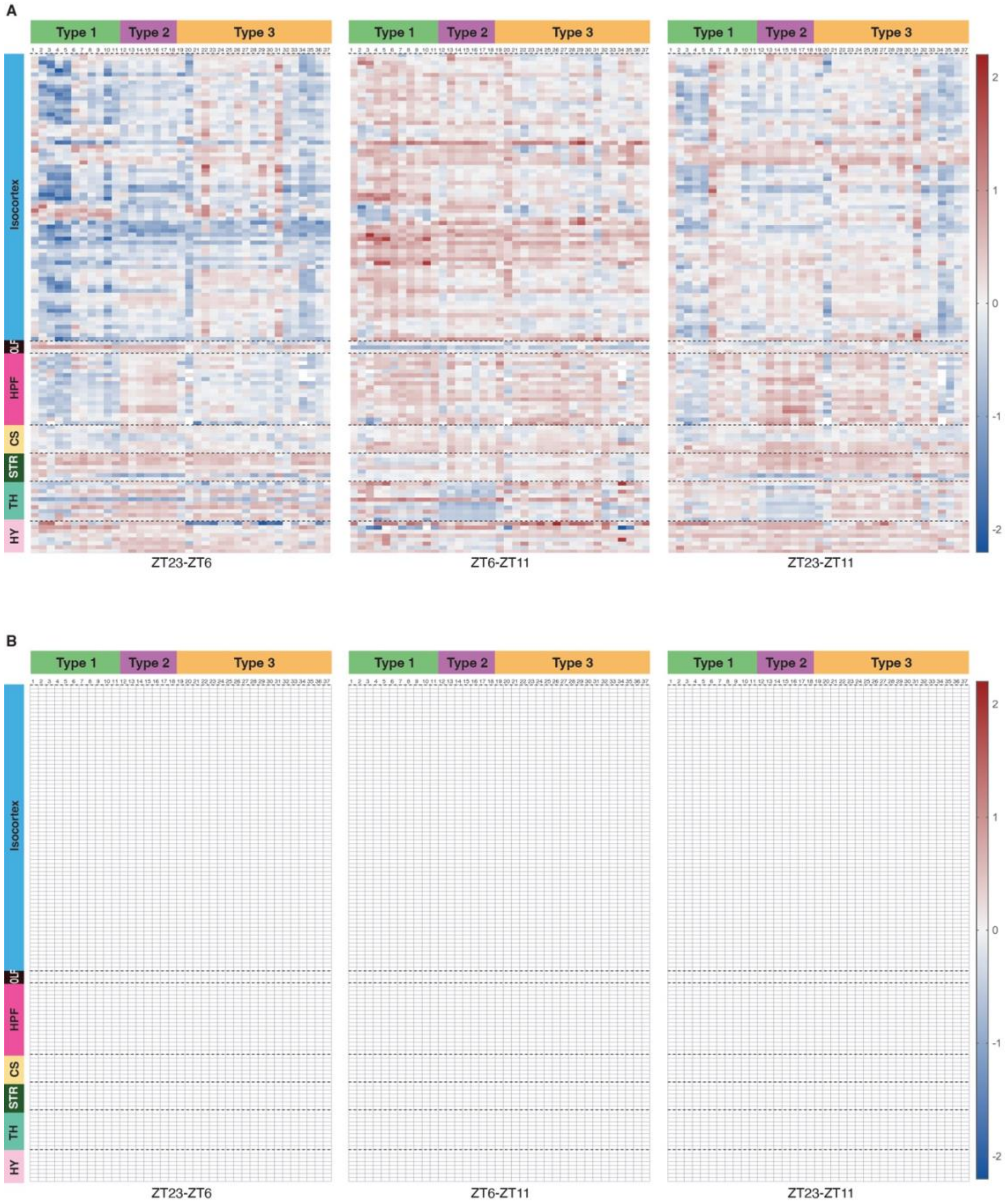

Figure S2 The synaptome architecture of Type 1, Type 2 and Type 3 synapse subtypes in the circadian cycle. Related to Figure 2.

Heatmaps of SD-induced changes in the density (Cohen's d) of synapse subtypes in brain regions for periods ZT23-ZT6 (left panel), ZT6-ZT11 (middle panel), ZT23-ZT11 (right panel). (A) raw values, (B) significant values after multiple correction testing (Bayesian test with Benjamini-Hochberg correction). All datapoints show P>0.05. Regions; isocortex; OLF, olfactory areas; HPF, hippocampal formation; CS, cortical subplate; STR, striatum; TH, thalamus; HY, hypothalamus.

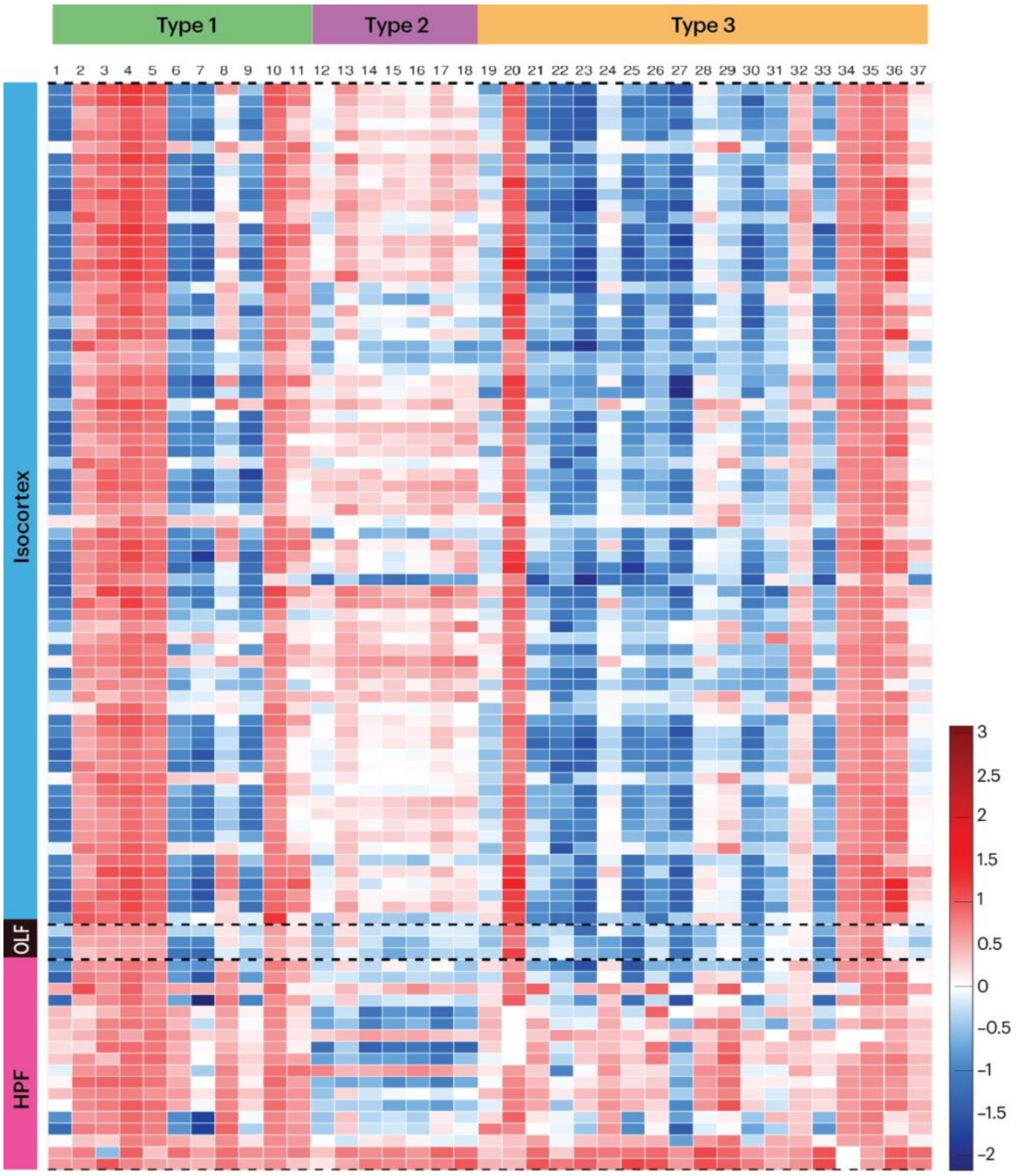

Figure S3. SD differentially impacts synapse subtypes. Related to Figures 2, 3. Heatmap of SD-induced changes in the density (Cohen's d) of synapse types and subtypes. Uncorrected. Significant changes are shown in Figure S4. Regions: isocortex; OLF, olfactory regions; HPF, hippocampal formation.

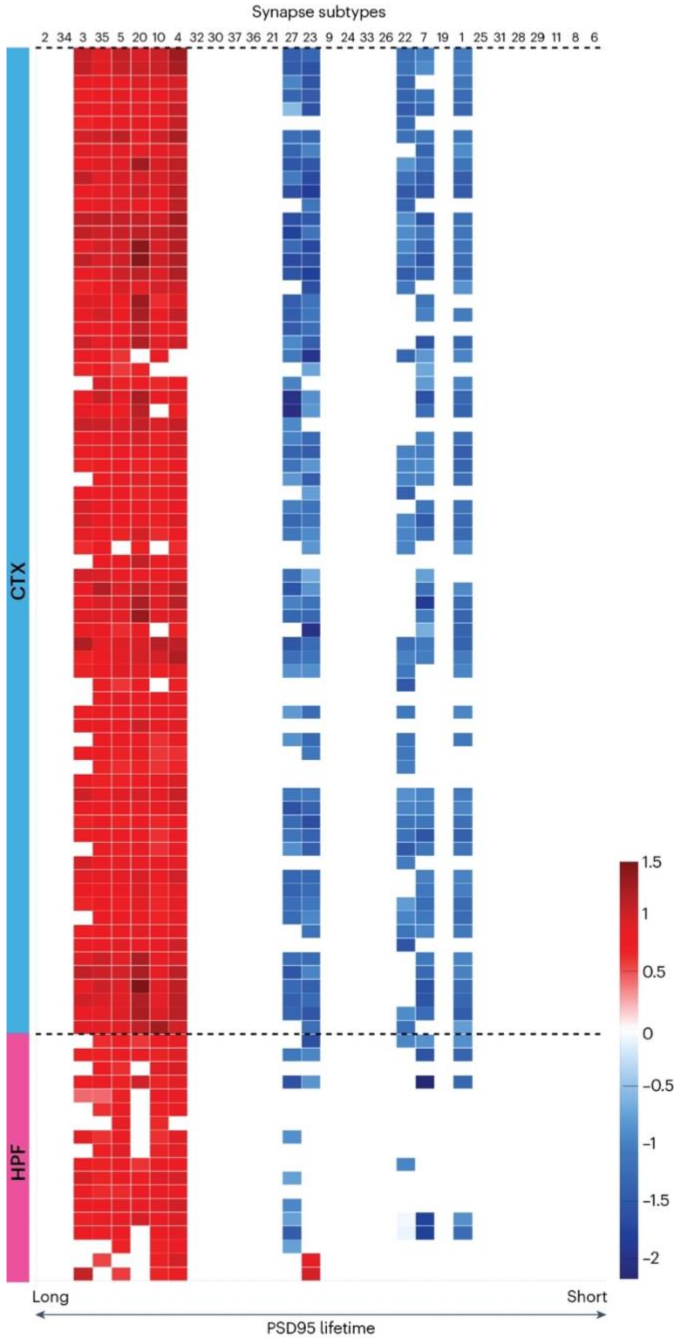

Figure S4. SD differentially impacts synapse subtypes with long and short protein lifetimes. Related to Figures 2, 3.

Heatmap of SD-induced changes in the density (Cohen's d) of synapse subtypes ranked from longest to shortest PSD95 lifetime[S1]. Significant changes are shown ($P<0.05$, Bayesian test with Benjamini-Hochberg correction).

Supplemental reference

S1. Bulovaite, E., Qiu, Z., Kratschke, M., Zgraj, A., Fricker, D.G., Tuck, E.J., Gokhale, R., Koniaris, B., Jami, S.A., Merino-Serrais, P., et al. (2022). A brain atlas of synapse protein lifetime across the mouse lifespan. Neuron *110*, 4057-4073 e4058.
